# Obesity medicine provider-directed health coaching in a commercial weight loss program: Proof of concept

**DOI:** 10.1016/j.obpill.2024.100146

**Published:** 2024-10-22

**Authors:** Michelle Alencar, Angela Fitch, Rachel M. Sauls, Kelly Johnson, Mayur Patel

**Affiliations:** a Department of Kinesiology, California State University Long Beach, Long Beach, CA, USA; b InHealth Medical Services, Inc., Los Angeles, CA, USA; c Knownwell, Boston, MA, USA; d Department of Nutrition, Gillings School of Global Public Health, University of North Carolina at Chapel Hill, Chapel Hill, NC, USA; e Department of Kinesiology, Coastal Carolina University, Conway, SC, USA; f California Chest and Medical Center, Burbank, CA, USA

**Keywords:** Diet, Health and wellness coaching, Lifestyle, Obesity, Weight reduction

## Abstract

**Background:**

Obesity remains a leading serious chronic disease and cause of death in the U.S. Despite previous research in diets (i.e., caloric restriction), physical activity levels, and biochemical markers, no studies to date have investigated the combination of an obesity medicine physician with a health coach alongside a commercial program for reducing body weight. As a result, this pilot study aims to assess the relationship between a commercial diet program and health and wellness coaching (HWC) for weight reduction outcomes when delivered in conjunction with an obesity medicine physician chronic disease care model, as a proof-of-concept study.

**Methods:**

This 24-week proof-of-concept study was pragmatic, being an unblinded, unrandomized, uncontrolled, longitudinal, observational study. Its purpose was to assess participant weight reduction from a commercial weight reduction program using a pre-packaged portion-controlled reduced-calorie diet while meeting with a health coach and obesity medicine physician for counseling and support. Participants in the sample underwent a commercial weight reduction program through dietary control while meeting with an HWC. A repeated measures ANOVA was conducted to determine the weight reduction trends across a 24-week program.

**Results:**

This study included n = 53 participants; a majority were white females (n = 45; 85 %), with an average (SD) age of 50.3 (12.2) years. There was a significant improvement in weight reduction (21.8 ± 9.4 lbs. WL) seen throughout the 24-week program (F (7, 364) = 33.129, p < 0.001), with a large effect (η^2^ = 0.922).

**Conclusion:**

This proof-of-concept project found that Obesity Medicine Physician-directed, portion-controlled meals and HWC can improve weight reduction across a 24-week program using portioned-controlled meals. Confirmation of these findings and their clinical significance requires a follow-up randomized, controlled clinical trial using validated assessment tools.

## Graphical abstract

**Figure:**
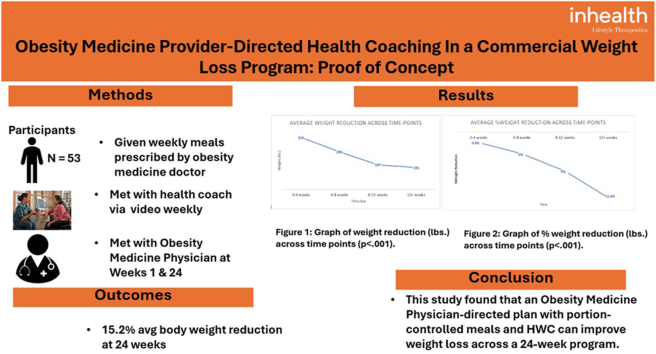

## 1 Introduction

### 1.1 Background and objective

Obesity is a disease that can dramatically heighten the susceptibility to other comorbidities, such as heart disease and diabetes, afflicting more than two-thirds of American adults [1,2]. Despite the extensive body of evidence dedicated to obesity research, the quest for sustained weight reduction and weight maintenance persists as a multifaceted challenge for individuals battling obesity. Within this complex landscape, numerous factors serve both as facilitators and formidable barriers in the enduring struggle for effective, long-term weight management [3]. According to current literature, caloric restriction, lifestyle behavior modification encompassing physical activity and dietary adjustments, and the judicious use of anti-obesity medications when deemed suitable offer auspicious pathways for achieving and maintaining weight reduction and effective weight management [4,5].

Caloric restriction reduces the number of calories ingested daily and is a method to maintain healthy weight reduction [6]. Considering the prevalent issue of caloric underestimation among individuals with obesity, where an astonishing 40 % of daily caloric intake tends to be inaccurately gauged through traditional dietary tracking methods, there has been a growing interest in innovative weight management strategies. Certain commercial brands have used portion-controlled entrees to support portion control. This method aims to support individuals in their weight reduction journey without necessitating the meticulous quantification of nutrients or the burdensome task of tracking their dietary intake [[7], [8], [9]]. Research has shown that portion-controlled meals can reduce under-reporting kcals and increase weight reduction outcomes [7]. Furthermore, the role of obesity medicine providers has grown significantly in importance, as they concentrate on aiding patients grappling with overweight and obesity, often making them more accessible to patients seeking care through primary care physicians rather than specialists [10,11]. However, a significant research gap persists in investigating the oversight of obesity-related healthcare by medical providers in tandem with a commercial weight reduction program. Numerous studies have underscored the importance of lifestyle and behavioral modifications in facilitating nutritional changes, including caloric restriction, increased physical activity, and adopting beneficial behaviors such as weight monitoring and diet tracking. These interventions have been shown to significantly improve weight reduction outcomes [5,12,13].

Consequently, a compelling movement has emerged advocating for integrating lifestyle interventions into broader healthcare strategies to combat the pervasive challenge of obesity. One promising avenue involves the collaboration between health and wellness coaching (HWC) and obesity medicine providers, offering a comprehensive approach to address this pressing public health issue [14].

HWC is a counseling style that focuses on improving individuals' health and well-being through empowering lifestyle behavioral changes (e.g., fitness, nutrition, stress coping, mindfulness) [15]. HWC has been linked to improved weight reduction outcomes through behavioral strategies learned from sessions, such as changes that match an individual's lifestyle for long-term adherence [16,17]. Previous qualitative research indicates that HWC is effective through personal rapport established by HWC to improve health outcomes [18]. Additionally, HWC allows individuals to make behavioral changes based on their distinct needs and preferences, improving the likelihood of long-term change [19,20]. Preliminary evidence indicates that HWC with physician oversight (e.g., appointments and monitoring) and controlled diets improve weight reduction and health outcomes [20]. However, further research is needed to better understand how HWC impacts weight reduction with nutritional maintenance (i.e., commercial program).

There is a critical need for effective long-term weight management strategies. Yet, there is a notable gap in research regarding the integration of HWC, portion-controlled nutritional programs, and obesity medicine provider-directed care. Our hypothesis suggests that such an integration holds promise for yielding sustained weight reduction over extended periods. This research addresses this gap and provides valuable insights into optimizing weight management approaches for improved patient outcomes. As a result, the primary purpose of this study was to investigate the interplay between a commercial diet program with portion-controlled nutrition, HWC, and obesity medicine provider care, assessing their collective impact on weight reduction outcomes over a 24-week program duration.

## 2 Methods

### 2.1 Study design

This 24-week pilot study was pragmatic, being an unblinded, unrandomized, uncontrolled, longitudinal, observational study to assess participant weight reduction from a commercial weight reduction program using a pre-packaged portion-controlled reduced-calorie diet while meeting with a health coach and obesity medicine physician for counseling and support. Participants in the sample underwent a commercial weight reduction program using a pre-packaged portion-controlled reduced-calorie diet while meeting with three trained and certified HWCs who also had at least a high school level education (inHealth Medical Services, Inc., Los Angeles, CA) [13,20]. They also met with an obesity medicine physician to direct their care. They did not utilize any anti-obesity pharmacotherapy. All data was collected through electronic health records and de-identified before analysis.

### 2.2 Ethical review

Before the study started, all patients verbally consented through the medical clinic and weight reduction program. Coastal Carolina University's Institutional Review Board reviewed the protocol, approved the informed consent process, and determined that due to the nature of this research, verbal consent of study participants was sufficient (Protocol #2021.96).

### 2.3 Program

Patients underwent a reduced calorie portion-controlled weight reduction program and met with an HWC weekly for the program duration (24 weeks). The weight reduction program provided weekly meals that fit a prescribed diet plan provided by the physician overseeing obesity medicine. Visits with a HWC were conducted weekly via videoconferencing, for an average of 30 min per session for the 24-week study. The structure of the health coaching sessions is presented in Table 1. Health education topics were related to healthy weight reduction, including healthy nutrition, calorie reduction, physical activity, and behavior modification.

**Table 1:** Health and wellness coaching program (HWC) content across the study program.

Timeline	Health and Wellness Coaching (HWC) sessions	Physician Sessions
Week 0	Weekly sessions (months 1–3)	Meets to collect anthropometrics (height and weight) and monitor health status
Week 1-4	Create and monitor progress toward 9-month wellness goals.	
Week 4-8	Discuss nutritional, physical activity, and progress towards goals. Build and maintain rapport.	
Week 8-12	Motivational interviewing to support generative moments and explore strengths and values to health behavior change	–
Week 12-24	Biweekly Sessions (months 4-6). Continue the above content.	Meets to collect anthropometrics and monitor health status

### 2.4 Variables

Biomarkers were collected weekly for analysis. Variables included age, gender (self-reported view of themselves), race, weight (via Bluetooth scale), and height. Researchers were provided with de-identified data before analysis. Additional variables collected include the number of HWC visits and duration in both programs.

### 2.5 Statistical analysis

Descriptive statistics were conducted for demographics and variables of interest (e.g., gender, age, weight, and height). A repeated measures ANOVA was performed to assess the relationship between weight reduction across the 24-week program and 4-week intervals (e.g., baseline, 4-week, 8 weeks, etc.). A Mann-Kendall correlation was conducted to determine the independent relationships of weight trajectories across the 4-week intervals of the 24-week program. The effect size was determined for each comparison to better understand the relationship across variables and the risk of error.

## 3 Results

### 3.1 Main findings

**Fig. 1:**
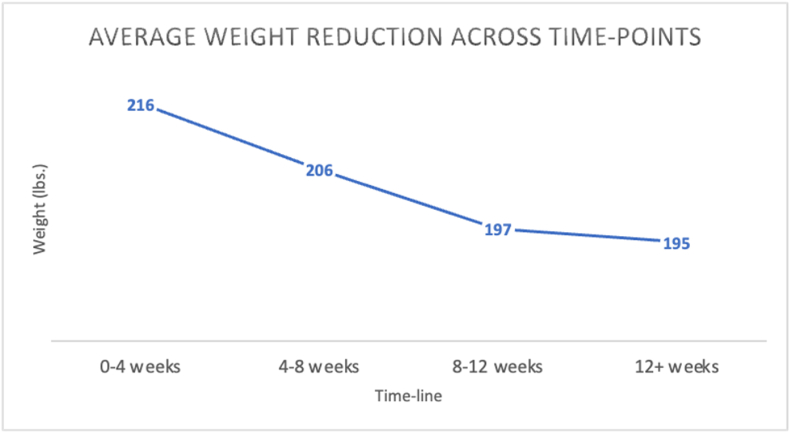
Graph of final weight (lbs.) across time points (p ​< ​0.001). ∗lbs ​= ​pounds.

**Fig. 2:**
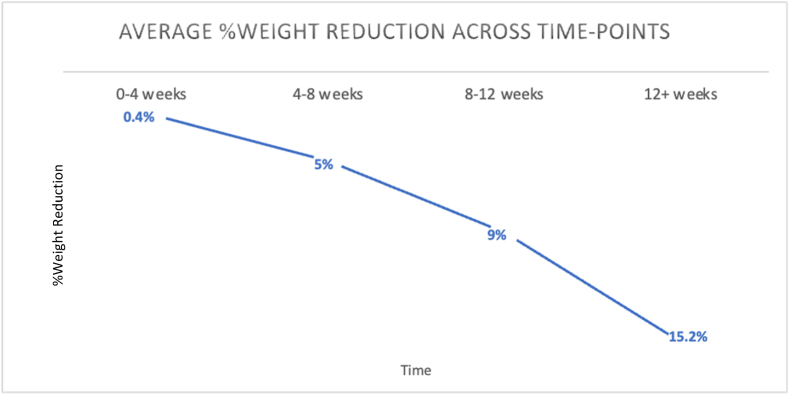
Graph of % weight reduction across time points (p < 0.001).

Our sample consisted of 53 participants, with a predominant female representation (n = 45; 85 %). The average (SD) age of the participants was 50.3 (12.2) years, and the majority identified as white/Caucasian (n = 47; 87 %). The initial average (SD) weight and BMI were 218.8 (40.3) lbs. and 35.8 (5.2) kg/m^2^, respectively. Detailed demographic information for all participants is provided in Table 2. The average (SD) weight reduction in this sample was 19.5 (5.2) lbs., and the average (SD) weeks of meeting with an HWC was 9 (3) weeks, as seen in Fig. 1. A repeated measures ANOVA was conducted to determine the relationship between time spent in the 24-week program and weight reduction. The means and standard deviations for weight reduction can be seen in Table 3. There was a significant increase in weight reduction seen across the 24-week program (F (7, 364) = 33.129, p < 0.001). Mauchly's test indicated that the assumption of sphericity had been met, (ꭓ^2^ (27) = 291.258, p < 0.001), and therefore degrees of freedom were corrected through Greenhouse Geisser estimates of sphericity (ε = 0.357). The effect of time in the 24-week program on weight reduction was large (η^2^ = 0.389). However, results from the Kendall-Mann test indicate that the greatest significant difference was seen between baseline (r = 0.257), week 12 (r = 0.589), and week 24 (r = 0.337; p < 0.001). A graphical representation of the results can be seen in Fig. 2.

**Table 2:** Participant characteristics. Note: N = 53 for the entire sample.

VARIABLE	MEAN ± bSD [RANGE]
AGE (YEARS)	50.4 ± 12.2 [26.0–76.0]
NUMBER FEMALE (%)	45 (84.9)
NUMBER WHITE (%)	42 (79.2)
aBMI	35.8 ± 5.2 [30.0–52.5]

a BMI = body mass index.

b SD = standard deviation.

**Table 3:** Weight reduction reported across 24-week program (p < 0.001).

Time-point	Mean (albs.)	bSD (albs.)	N-size
Baseline	0	0	53
4 weeks	0.5	4.1	53
8 weeks	5.2	3.2	53
12 weeks	10.2	6.0	53
16 weeks	15.6	7.8	53
20 weeks	18.7	10.5	50
24 weeks	21.8	9.4	40

a lbs. = pounds.

b SD = standard deviation.

## 4 Discussion

### 4.1 Overview

Obesity has surged to the forefront as a paramount chronic disease and a critical healthcare concern in the United States, necessitating research with an unwavering focus to discern lasting solutions, by experts in obesity medicine [21]. This pilot study was designed to better understand the relationship between HWC and a commercial diet program with obesity medicine physician oversight on weight reduction over time. The findings of this study support the idea that HWC, in conjunction with a commercial weight reduction program, significantly improves weight reduction over time when combined with an obesity medicine provider's medical oversight. The results convey that weight reduction significantly improved across the 24-week program while meeting both with a coach and obesity medicine physician and adhering to a commercial portion-controlled nutrition program. The results of this study offer compelling and promising insights into the effectiveness of innovative weight reduction programs that combine commercial-based diet regimens with health coaching.

### 4.2 Implications

Despite research developments, weight reduction maintenance has been frequently reported as challenging with several barriers (e.g., social, environmental, biological). Additionally, data suggests weight trajectories can fluctuate throughout weight reduction programs due to previously mentioned barriers [22,23]. However, the results from this study indicate that HWC with a portion-controlled nutrition program reduces the risk of weight fluctuations and provides more weight reduction over time. The findings support previous research on HWC and weight change, with a systematic review finding that HWC can improve weight reduction [24]. Furthermore, this pilot study's findings show that individuals who stayed in the program longer had greater weight reductions, supporting current research that long-term behavioral interventions have improved weight reduction outcomes [5]. However, future research is needed to understand the long-term relationship between HWC and weight changes (i.e., > one year) [25].

Portion-controlled meals and caloric restriction have promising findings in weight reduction over time, as seen in previous studies [[26], [27], [28]]. These meals offer precision regarding caloric restriction to reduce the risk of overeating or incorrect measurements [27,28]. However, another study found no significant weight reduction difference from the usual advice, potentially indicating that an HWC improved diet adherence and WL outcomes [29,30]. In comparison to obesity medications (e.g., liraglutide, phentermine, and bupropion/naltrexone), there are similar weight reduction outcomes [31]. Previous studies indicate that after 24-weeks of taking anti-obesity medications (E.g. lorcaserin), patients have similar weight reduction outcomes (e.g., 18 lbs. WL among n = 1538) [32]. The findings of this study provide promising results related to weight reduction, without the risk of adverse side effects that may result from some anti-obesity medications, such as nausea, constipation, and diarrhea [31,33]. This type of portion-controlled meal program could also be used in combination with less effective (and less costly) anti-obesity medications to produce greater weight reduction in combination with medicines as another potential future research opportunity.

### 4.3 Limitations

Although the findings of this study offer promising insights into the dynamic trajectories of weight over time, it is essential to acknowledge several inherent limitations associated with gender bias, sample size constraints, and study design. Also, this study was an unrandomized, uncontrolled, longitudinal, observational study, which could lead to biased results, inability to compare to controls, and potential overestimation of favorable results. The study predominantly comprised white women around the age of 50, which does not adequately represent the broader population. Future research endeavors must prioritize validating these findings across diverse ethnicities and genders. Additionally, the relatively small sample size in this study (n = 53) underscores the need for larger-scale investigations to assess the feasibility and effectiveness of HWC in effecting sustained changes in weight over time. It is noteworthy that a review of existing literature has revealed a recurrent issue of limited sample sizes, with most behavioral interventions employing HWC featuring participant cohorts under 60, which may impede the robustness and generalizability of study results [35]. Addressing these limitations in future research will help provide a more comprehensive and robust understanding of the potential impact of HWC on diverse populations and enhance the validity of study outcomes [34]. Additionally, future studies are needed to determine if HWC can improve physical activity levels, self-care strategies, or stress management skills. Although this study utilized a 24-week program, previous research suggests that behavioral interventions should assess changes up to a year post-intervention to determine adherence and long-term behavioral changes.

## 5 Conclusion

In conclusion, this study presents compelling evidence supporting the effectiveness of HWC in enhancing weight reduction outcomes throughout a 24-week program, particularly when integrated with the oversight by an obesity medicine provider. These findings hold promise, shedding light on the potential for behavioral interventions involving HWC to yield enduring and consistent results, minimizing fluctuations that often hinder long-term success. Nevertheless, the path forward beckons us to delve deeper into the intricate relationship between HWC and other crucial facets of a healthy lifestyle, such as physical activity and self-care. We can uncover the full spectrum of possibilities through continued exploration, enriching our understanding and empowering individuals on their transformative journeys toward improved health and well-being.

## Author contributions

All authors contributed to the manuscript. MA developed the project idea, attained data, and wrote and reviewed the manuscript. AF reviewed and edited the manuscript. RS completed the data analysis and wrote and reviewed the manuscript. KJ wrote and reviewed the manuscript. All authors reviewed and approved of the final submitted version.

## Disclosure

Angela Fitch: Eli Lilly, Jenny Craig, Novo Nordisk, SideKick, Seca, Vivus, Currax, Carmot advisory boards.

Michelle Alencar and Kelly Johnson have stock ownership in inHealth Medical Services, Inc.

All other authors have no disclosures or competing interests.

## Key takeaways

• This pilot, proof-of-concept uncontrolled study suggests that Obesity Medicine Physician-directed with portion-controlled meals and HWC may improve weight reduction scores across a 24-week program using portion-controlled meals. Confirmation of these findings and their clinical significance requires a follow-up randomized, controlled clinical trial using validated assessment tools.
• Health and wellness coaching (HWC), when combined with oversight by an obesity medicine provider, may improve weight reduction, with the potential for consistent and lasting results, reducing the fluctuations that typically impede long-term success
• Further research is needed to explore the relationship between HWC and other key aspects of a healthy lifestyle, such as physical activity and self-care, to understand and fully maximize its benefits.

## Declaration of use of artificial intelligence

No use of artificial intelligence was used in the writing of this manuscript.

## Funding

No funding was acquired for this manuscript.
